# Melanoma diagnosis using deep learning techniques on dermatoscopic images

**DOI:** 10.1186/s12880-020-00534-8

**Published:** 2021-01-06

**Authors:** Mario Fernando Jojoa Acosta, Liesle Yail Caballero Tovar, Maria Begonya Garcia-Zapirain, Winston Spencer Percybrooks

**Affiliations:** 1 eVida Research Laboratory, University of Deusto, Avda. Universidades 24, 48007 Bilbao, Spain; 2 Department of Electrical and Electronics Engineering, Universidad del Norte, Km.5 Vía Puerto Colombia, Barranquilla, Colombia

**Keywords:** Mask R_CNN, Deep learning, Transfer learning, Convolutional neural network, Object detection, Object classification

## Abstract

**Background:**

Melanoma has become more widespread over the past 30 years and early detection is a major factor in reducing mortality rates associated with this type of skin cancer. Therefore, having access to an automatic, reliable system that is able to detect the presence of melanoma via a dermatoscopic image of lesions and/or skin pigmentation can be a very useful tool in the area of medical diagnosis.

**Methods:**

Among state-of-the-art methods used for automated or computer assisted medical diagnosis, attention should be drawn to Deep Learning based on Convolutional Neural Networks, wherewith segmentation, classification and detection systems for several diseases have been implemented. The method proposed in this paper involves an initial stage that automatically crops the region of interest within a dermatoscopic image using the Mask and Region-based Convolutional Neural Network technique, and a second stage based on a ResNet152 structure, which classifies lesions as either “benign” or “malignant”.

**Results:**

Training, validation and testing of the proposed model was carried out using the database associated to the challenge set out at the 2017 International Symposium on Biomedical Imaging. On the test data set, the proposed model achieves an increase in accuracy and balanced accuracy of 3.66% and 9.96%, respectively, with respect to the best accuracy and the best sensitivity/specificity ratio reported to date for melanoma detection in this challenge. Additionally, unlike previous models, the specificity and sensitivity achieve a high score (greater than 0.8) simultaneously, which indicates that the model is good for accurate discrimination between benign and malignant lesion, not biased towards any of those classes.

**Conclusions:**

The results achieved with the proposed model suggest a significant improvement over the results obtained in the state of the art as far as performance of skin lesion classifiers (malignant/benign) is concerned.

## Background

By the year 2019, the American Cancer Society (ACS) has calculated that approximately 96,480 new cases of melanomas will be diagnosed and that approximately 7230 people will die from melanoma in the United States [1]. Melanoma is considered to be the most serious type of skin cancer, meaning it is particularly important to accurately diagnose it as soon as possible, to allow treatment before any dissemination and/or metastasis, as this will significantly increase the chance of recovery [1–4]. Generally speaking, melanoma can be recognized via a visual inspection that focuses on the region of the cutaneous lesion. However, there is great similarity between melanomas and other skin lesions such as nevus, increasing the difficulty in performing cancer classification and diagnostic tasks in skin lesions [5]. Therefore, ensuring access to a reliable, practical system that is able to conduct an automated evaluation of a skin lesion would have a positive influence on patient care and increases the chance of early detection of cancer, thus reducing mortality rates associated with this disease.

In dermatology, both diagnosis and monitoring of skin lesions have relied mainly on visual inspection and other non-invasive evaluations, invasive procedures are avoided because they can destroy the lesions and make it impossible to carry out a clinical monitoring of its evolution [6]. In highly selected patient populations, computer assisted diagnosis methods have demonstrated high sensitivity and may be useful as a back-up diagnostic aid for specialists to reduce the risk of missing melanomas [7]. One of the non-invasive methods employed in diagnosis and monitoring is the use of confocal microscopes available for clinical use, which provide sharp images because they capture only the light from the plane of the sample in focus. In vivo reflectance confocal microscopy captures high resolution images in real time and is used in the evaluation of various dermatological conditions [6]. Reflectance confocal microscopy may reduce unnecessary excisions without missing melanoma cases [8]. For its part, research results in [9] show that dermoscopy is more accurate than visual inspection alone; the results reported in [9, 10] indicate that melanomas would not be detected using visual inspection. In our proposed model, the conclusive analysis of dermatoscopic images is automated through artificial intelligence algorithms, obtaining a preliminary characterization that serves as support for dermatologists in order to obtain faster and more precise diagnoses.

Deep automatic models base their learning on the samples used for their training, this work uses the ISIC 2017 database, which has more than 2000 high resolution dermatoscopic images grouped into 3 main categories: Melanoma, nevus and keratosis. Testing determines if these data is enough to achieve high performance detecting and rejecting benign and malignant dysplasic lesions.

From a technical standpoint of the problem, it has been found that many cutaneous lesion detectors have been proposed using Deep Learning models based on Convolutional Neural Networks (CNN), such as GoogLeNet-AlexNet-ResNet-VGGNet ensembles [11], R_CNN [12], the Mask and Region-based Convolutional Neural Network (Mask R_CNN) and DeeplabV3+ method ensemble [13] and in general many CNN structures, making this approach one of the most powerful for effective feature extraction and classification [14]. The method proposed in this work exploits the potentialities of Mask R_CNN for detection of objects, patterns and/or figures within an image together with potentiality of the ResNet152 model for classification purposes. The ResNet152 model was selected after testing with other ResNet, Inception and VGG structures, all of them with demonstrated high-performance on several image classification tasks [15, 16].

The main factors that make classification of dermatoscopic images difficult are: the presence of hairs, inks, ruler markings, colored patches, glimmers of light, drops, oil bubbles, blood vessels, hypopigmentation areas and/or inflammation around the lesion, among others [17]. Extraction features in CNN is affected when this undesired noise is present in the image, directly affecting performance of the classifier. Therefore, accurate extraction of the region of interest (ROI) is considered an essential step towards improving performance of the system [18, 19]. To solve the melanoma detection problem, taking into account the issues mentioned above, we propose a two-stage classification method:

- Stage 1: Use Mask R_CNN to create a bounding box around the skin lesion, with as few visual noise as possible. The output of this stage is the cropped bounding box.
- Stage 2: Classification of the cropped area using ResNet152. Although Mask R_CNN can also be used for image classification, not only for object detection as in Stage 1, we decided to separate the two tasks in order to be able to use higher performing classification models.

Something that should be stressed in this work is the constant search for high-performance models using different balancing ratios for the training data used by the algorithms. This is of great interest for the scientific community, given that unbalanced repositories are very common in the medical field.

The most recently published dermatoscopic image classification work (malignant/benign) for the ISIC (International Skin Imaging Collaboration) 2017 challenge, using the data set from the International Symposium on Biomedical Imaging (ISBI) 2017 challenge, is taken as a reference within the reviewed state-of-the-art. An objective comparison of results is made with our proposed method using the evaluation metrics adopted by the challenge such as specificity, sensitivity and accuracy. However, we added another metric—balance accuracy—suggested for this particular case in which there is an unbalanced database [20–22]. A Receiver Operating Characteristic (ROC) space is provided in order to compare and display performance of various classifier models in a single graph, and this is a very useful metric for decision-making in the medical field, the use of which has been increasing in machine learning research [23].

The rest of this paper is organized as follows: "Methods" section explains in detail the proposed method; "Results" section shows the results obtained from the experiments carried out to select the best performing model for the proposed method and compares the best results obtained from the ISIC 2017 challenge with the proposed method; "Discussion" section provides a discussion and finally, "Conclusions" section explain the conclusions.

## Methods

**Fig. 1:**
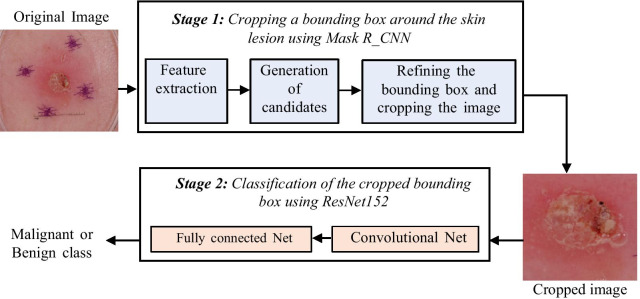
Functional block diagram of the proposed system

We propose an automated classification method for cutaneous lesion in digital dermatoscopic images, in order to detect the presence of melanoma. This method comprises two fundamental stages, which are: Stage 1: Cropping a bounding box around only the skin lesion in the input image, using Mask R_CNN; and Stage 2: Classification of the cropped bounding box using ResNet152, as described in Fig. 1 with a functional block diagram of the system.

In the first stage, Mask R_CNN makes a selection of candidates, i.e. it filters regions within the image that may contain a skin lesion in order to be classified. The main purpose of this step is to crop a bounding box of the image around the region of interest (ROI). We then train, validate and test the model in this stage using the dataset from the ISIC 2017 challenge [24], which contains 1995 dermatoscopic images with their respective masks for training, 149 dermatoscopic images for validation and 598 dermatoscopic images for testing [25].

**Fig. 2:**
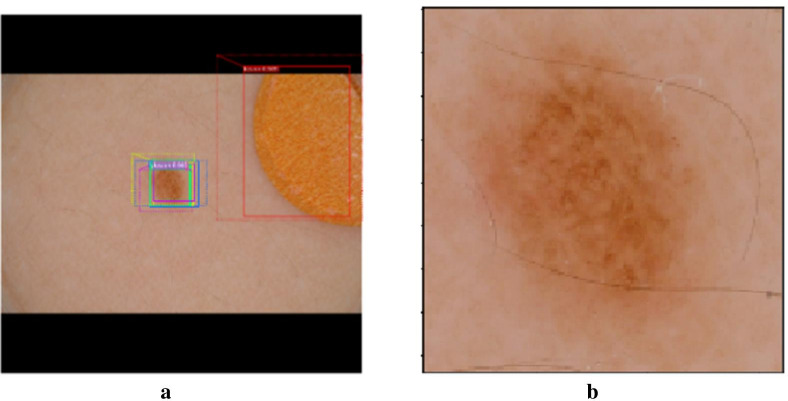
**a** ROIs after refining; **b** Cropped image

Mask R_CNN trains the system with masks provided by a clinical expert, so as to be able to identify those pixels that correspond to a skin lesion within the image, i.e. our region of interest (ROI). Although there are many candidates in an image, calculating the probability that one candidate belongs to a skin lesion plays a major role when rejecting other elements from the image such as marks, patches or bubbles, among others. Mask R_CNN takes candidates and classifies them by generating probabilities as shown in Fig. 2a, in which the area corresponding to the yellow patch present in the image has a lower probability of being a skin lesion (0.505 probability) than the probability calculated in a second region that genuinely corresponds to a skin lesion (0.982 probability). Lastly, we crop the area defined by the refined bounding box and create a new image Fig. 2b that is then classified on Stage 2.

In Stage 2, in order to classify the cropped images identified as skins lesions from Stage 1, we decided to include a ResNet152 classifier that outputs a benign or malignant label. A “malignant” class being understood as referring to those skin lesions identified as melanoma and “benign” class referring to all those lesions not identified as melanoma.

**Fig. 3:**
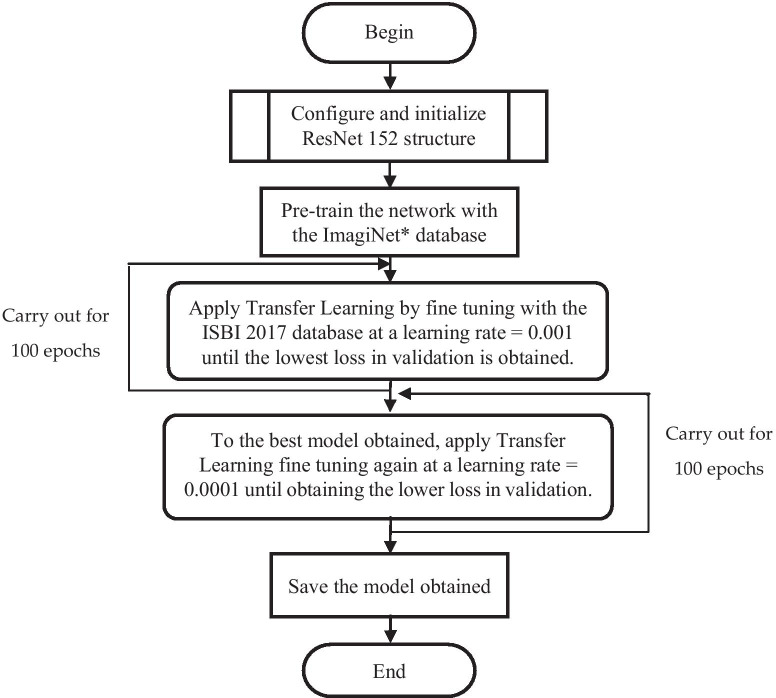
Flowchart of the network training process. *ImagiNet database [26]

Figure 1 also shows the generic internal structure of ResNet152, which comprises two blocks: one containing the feature extraction layers known as convolution layers, and the other performing the classification via a fully connected network. In overall terms, this convolutional network is made up of over 60 million parameters that need to be adjusted in a process known as training, which usually requires a very large volume of images. The relatively small amount of data available in the ISBI 2017 database (1995 items for training and 149 for validation) makes it unsuitable to train such a large network from scratch. Therefore, we decided to use the technique known as Transfer Learning, which uses a network pre-trained using a very large general-purpose database, and subsequently retrains it using an smaller, specific-purpose database. In our case, this was undertaken following the steps shown in Fig. 3.

An iterative adjustment of the learning rate [27] is undertaken following the procedure described in Fig. 3 for the five training scenarios listed in Table 1, in which the ratio between the number of malignant and benign dermatoscopic images is modified.

**Table 1:** Five different training scenarios

Model	Technique to modify the malignant/benign training ratio	Malignant/Benign training ratio
Model 1	None	0.231
Model 2	Data augmentation applied to “malignant” class images, no changes to “benign” class images	0.440
Model 3	Data augmentation applied to “malignant” class images, down sampling applied to “benign” class images	0.463
Model 4	Data augmentation applied to “malignant” class images, down sampling applied in “benign” class images	1
Model 5	Data augmentation and addition of PH2 images applied to “malignant” class images, no changes to “benign” class images	0.256

The original dataset from the ISBI 2017 challenge was used to obtain model 1, unlike models 2, 3, 4 and 5, in which we expanded the size of the “malignant” class training data set by 90% by generating modified versions of the dermatoscopic images and applying the following data augmentation techniques: rotation augmentation (image rotation by 180 degrees) and vertical flip augmentation (pixel reordering by row reversal). In models 3 and 4, we also reduced the number of dermatoscopic images from the “benign” class by different ratios. Lastly, in model 5 we included dermatoscopic images with “malignant” skin lesions extracted from the PH2 database [28].

The only hyper-parameters adjusted were the learning rate and the number of epochs, with the best performing values for each training scenario shown in Table 2. The momentum and batch size are set at 0.9 and 32 respectively.

**Table 2:** Adjustment of hyper-parameters

Model/Hyper-parameters	Number of epochs with learning rate 0.001	Number of epochs with learning rate 0.0001	
Model 1	79	87	
Model 2	85	95	
Model 3	67	90	
Model 4	93	74	
Model 5	56	59	

**Fig. 4:**
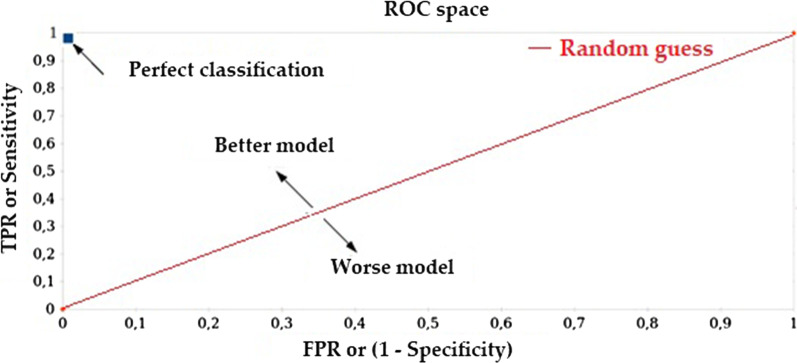
Receiver operating characteristic ROC space

For our application, the ResNet152 classifies between “malignant” or “benign” classes, thus resulting in a binary classifier whose performance is evaluated using specificity, sensitivity, accuracy and balance accuracy metrics, in which the reference class is the “malignant” class. Sensitivity and specificity are the standard metrics used to evaluate performance of classifiers in the field of medicine [13]. In the case of our skin lesion classifier, a specificity (Eq. (1)) close to 1.0 indicates good performance when correctly classifying the “benign” class. Conversely, a sensitivity (Eq. (2)) close to 1.0 indicates good performance when correctly classifying the “malignant” class (melanoma lesion). Accuracy (Eq. (3)) is another very commonly-used evaluation metric in the area of medical applications that measures the overall performance of a classifier [11–13, 17]; however, this metric is not suitable when the data set is highly unbalanced, as a classifier that is biased towards the most frequently occurring class leads to an optimistic estimate that would provide misleading results. This problem can be addressed by replacing this metric with a different one known as balanced accuracy for the specific case of a binary-type classifier [20, 21]. Specificity, sensitivity, accuracy and balanced accuracy of classifiers are calculated from the resulting confusion matrices, and also compared in a Receiver Operating Characteristic (ROC) space, as shown in Fig. 4.

A singular point in the ROC space is better than another if the latter is located closer to the northeast corner than the first, i.e. the coordinate (0,1) represents a perfect classifier. The ROC space is commonly used for decision-making in the field of medicine, as it shows the relative compensation that may exist between benefits (true positives) and costs (false positives) [23].

## Results

This section shows the results obtained when applying proposed method to the five training scenarios described in Table 1, in which a range of data transform techniques are used to reduce the degree of data imbalance during training.

**Fig. 5:**
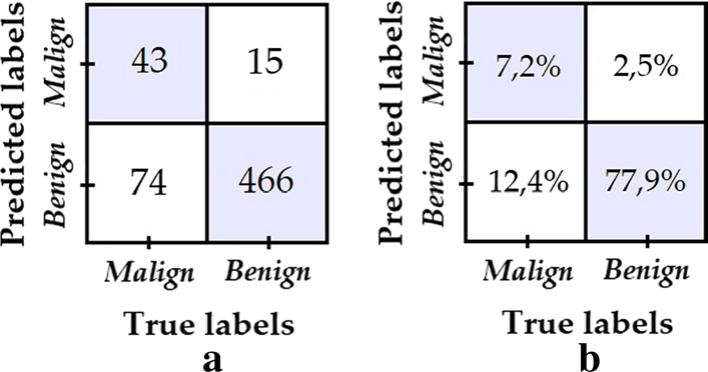
**a** Confusion matrix of model 1; **b** Percentage confusion matrix of model 1

Model 1 was obtained by training the classifier with the data set from the ISBI 2017 challenge, which contains 1995 dermatoscopic images for training, of which 1620 correspond to benign lesions and 375 to malignant lesions; as well as 149 dermatoscopic images for validation purposes, 119 corresponding to benign lesions and 30 to malignant lesions. Model 1 was trained without applying any augmentation technique or reduction data, obtaining the confusion matrix shown in Fig. 5 when tested with the test data from ISBI 2017 challenge, which comprises 598 dermatoscopic images, 481 corresponding to benign lesions and 117 to malignant lesions. All testing results we provide in this section for the five models were obtained over this same test data set.

Despite the fact that excellent performance is noted when classifying the “benign” class, i.e. high specificity, the same behaviour is not observed when classifying the “malignant” class, i.e. low specificity. A possible fundamental reason for this is the clear bias in the training data set towards the “benign” class. This reason, justifies the use of data augmentation/reduction techniques in order to reduce the training imbalance ratio and achieve better overall results, as it is explored with models 2–5.

**Fig. 6:**
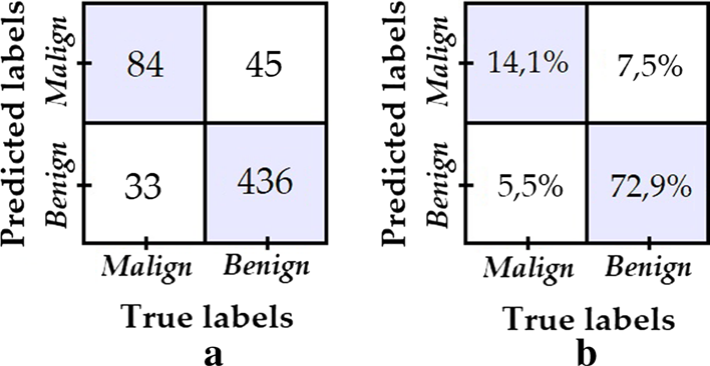
**a** Confusion matrix of model 2; **b** Percentage confusion matrix of model 2

To obtain model 2, a 90% transform augmentation is applied on the dermatoscopic images from the “malignant” class, thus increasing the balance ratio between the two classes from 0.231 to 0.440. The test data same as before is used to evaluate model 2, obtaining the confusion matrix shown in Fig. 6.

The number of “malignant” test samples that are correctly classified improves from 43 to 84 with respect to model 1. While there is also a drop in the number of “benign” samples correctly classified, it is not nearly as significant, therefore model 2 is postulated as a better model since it achieves a better balance in specificity versus sensitivity than model 1.

**Fig. 7:**
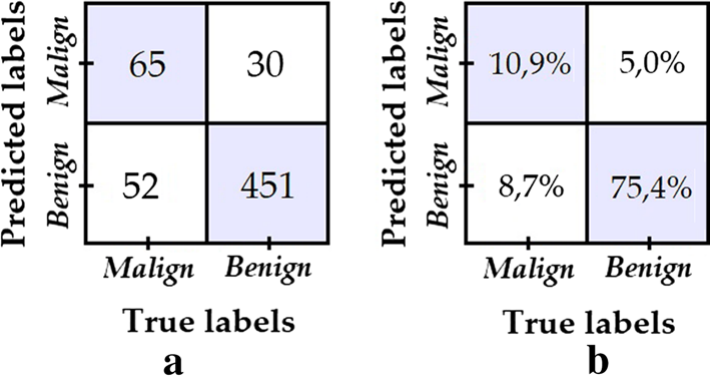
**a** Confusion matrix of model 3; **b** Percentage confusion matrix of model 3

In model 3, in addition to the same data augmentation procedure used in model 2 for the “malign” class, data down sampling is applied to the “benign” class images in order to increase the training balance ratio to 0.463. The confusion matrix shown in Fig. 7 was obtained from the evaluation of model 3.

We can see that despite the fact that the degree of data imbalance is lower, the number of “malignant” class images that had been correctly classified failed to improve with respect to model 2—rather, the opposite was true, it decreased.

**Fig. 8:**
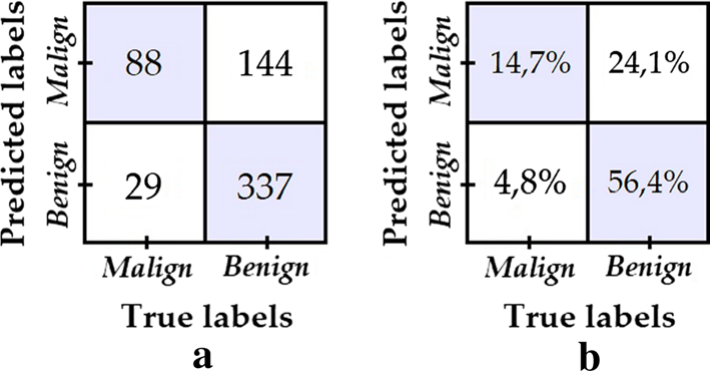
**a** Confusion matrix of model 4; **b** Percentage confusion matrix of model 4

Model 4 is obtained in the same way as model 3, but the down sampling and augmentation transforms are adjusted to a 1.0 training balance ratio, i.e. until we get the same number of malignant and benign training images. The confusion matrix shown in Fig. 8 was obtained from the test data set.

Despite the fact that the training data is perfectly balanced, the number of correctly classified images from the “benign” class was reduced considerably.

**Fig. 9:**
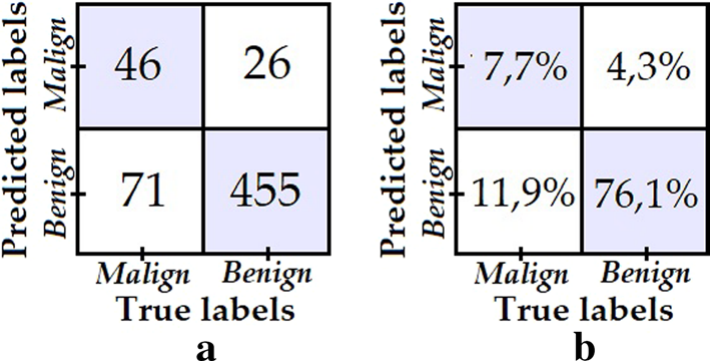
**a** Confusion matrix of model 5; **b** Percentage confusion matrix of model 5

Lastly, for model 5 data augmentation for the “malign” class was performed using data from the PH2 data set [28], while no changes were made to the “benign” class data. This resulted in a 0.256 training balance ratio, i.e. a minimum increase with respect to the original situation used for model 1. The confusion matrix obtained is shown in Fig. 9.

Despite the fact that new images belonging to the “malignant” class from a new dataset are added, no improvement in classification of “malignant” class images is noted.

**Fig. 10:**
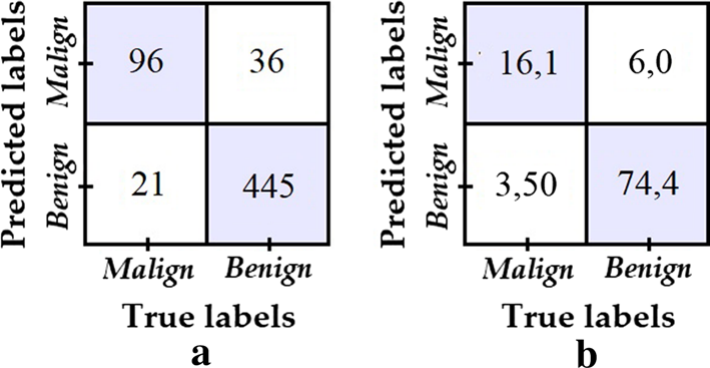
**a** Confusion matrix of model 6; **b** Percentage confusion matrix of model 6

Lastly, the best model from the previous tests (i.e. model 2) was retrained for 60 more epochs and a lower learning rate of 0.0001. The resulting model is called model 6 and its confusion matrix is shown in Fig. 10. Meanwhile, Table 3 summarizes the results obtained for all 6 tested models, where it is clear that, overall, model 6 shows the best performance.

**Table 3:** Result of evaluation metrics for the six models

Evaluation metric	Model 1	Model 2	Model 3	Model 4	Model 5	Model 6
Specificity	**0.968**	0.906	0.938	0.701	0.946	0.925
Sensitivity	0.367	0.718	0.556	0.752	0.393	**0.820**
Accuracy	0.851	0.870	0.863	0.711	0.838	**0.904**
Balanced accuracy	0.668	0.812	0.747	0.726	0.670	**0.872**

Bold values indicate the most relevant and representatives in this rearch work

Table 4 compares the results of the proposed model (model 6 from the previous section, now called eVida) against the best performing models reported for the ISIC challenge 2017 for melanoma classification. For the purposes of comparison, the table has been sorted from highest to lowest according to the reported Area Under the RC Curve (AUC) value, since AUC is the metric chosen by the organizers to define the ranking in this challenge. The challenge not only considers the AUC for melanoma detection, but computes an overall score that averages the AUC for melanoma detection with the AUC for keratosis detection. In this research work, we only consider the AUC for melanoma detection, since no keratosis tests were performed.

**Table 4:** Results of the proposed model versus models reported for the 2017 ISBI Challenge [29]

Organization	ACC_M	AUC_M	SE_M	SP_M	Balance accuracy	Overall score
eVida (proposed method M6)	**0.904**	**0.872**	0.820	0.925	**0.872**	**0.848**
RECOD Titans	**0.872**	**0.874**	0.547	0.950	0.749	0.792
Popleyi	0.858	0.870	0.427	0.963	0.695	0.762
Kazuhisa Matsunaga	0.828	0.868	0.735	0.851	**0.793**	**0.798**
Monty python	0.823	0.856	0.103	0.998	0.551	0.687
T D	0.845	0.836	0.350	0.965	0.658	0.726
Xulei Yang	0.830	0.830	0.436	0.925	0.681	0.708
Rafael Sousa	0.827	0.805	0.521	0.901	0.711	0.727
x j	0.843	0.804	0.376	0.957	0.667	0.710
Cristina Vasconcelos	0.830	0.791	0.171	0.990	0.581	0.660
Cristina Vasconcelos	0.825	0.789	0.171	0.983	0.577	0.658
Euijoon Ahn	0.805	0.786	0.009	0.998	0.504	0.614
Balázs Harangi	0.828	0.783	0.470	0.915	0.693	0.701
Matt Berseth	0.822	0.782	0.222	0.967	0.595	0.652
INESC Tecnalia	0.480	0.765	0.906	0.377	0.642	0.601
Dylan Shen	0.832	0.759	0.308	0.959	0.634	0.663
Vic Lee	0.832	0.757	0.308	0.959	0.634	0.665
Masih Mahbod	0.732	0.715	0.402	0.812	0.607	0.610
Dennis Murphree	0.760	0.684	0.231	0.888	0.560	0.574
Hao Chang	0.770	0.636	0.103	0.932	0.518	0.541
Jaisakthi S.M	0.748	0.623	0.419	0.828	0.624	0.614
Wenhao Zhang	0.805	0.500	0.000	1.000	0.500	0.581
Wiselin Jiji	0.503	0.495	0.470	0.511	0.491	0.433
Yanzhi Song	0.723	0.475	0.068	0.882	0.475	0.467

Bold values indicate the most relevant and representatives in this rearch work

*ACC* accuracy, *AUC* area under the RC curve, *SE_M* sensitivity, *SP_M* specificity for melanoma detection

The comparison between the proposed eVida model and the results reported by the RECOD Titans team (participant with the best previously reported AUC and ACC scores for melanoma detection) suggests an advantage for our proposed model since there is an increase in accuracy and balanced accuracy of 3.66% and 9.96% respectively for the test data. Meanwhile the reported AUC for both models is not significantly different. The proposed model also shows improvement over what is reported by Kazuhisa Matsunaga (Rank 1 in overall Lesion Classification), whose model presents the best balance between sensitivity and specificity among the previously reported results in melanoma detection for this challenge. Unlike the other reported models, in the proposed eVida model the specificity and sensitivity are both improved simultaneously to a high value (greater than 0.8), which indicates that the model is good for the detection of melanoma and non-melanoma (fewer overall errors). The metric called overall score in Table 4, which is defined as the average of the ACC, AUC, SE_M, and SP_M metrics, evidences the advantage of the eVida model over the previously reported models.

**Fig. 11:**
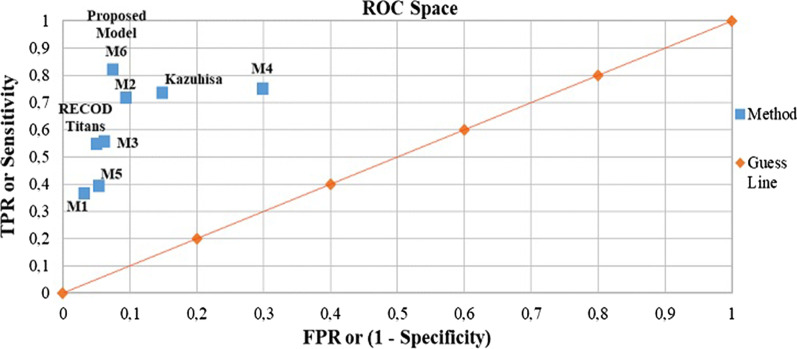
ROC space

Figure 11 shows a ROC space in which the five models tested in this work are located with labels M1, M2, M3, M4, M5 and M6. The models proposed by Recod Titans and Kazuhisa Matsunaga are also located in the figure.

Bearing in mind that the best model is the one whose singular point in the ROC space is found closest to the coordinate (0, 1), where the perfect classifier is located, we then measured these Euclidean distances and the results listed in Table 5 were obtained, arranged from lower to higher. From those values it can be concluded that the best classifier is eVida M6.

**Table 5:** Distance between the singular point of the model and the coordinate (0, 1) of the perfect classifier

Rank	Model	Distance between model and coordinate (0, 1)	
1	Model 6	0.195	
2	Model 2	0.297	
3	Kazuhisa Matsunaga	0.304	
4	Model 4	0.388	
5	Model 3	0.448	
6	Recod Titans	0.456	
7	Model 5	0.609	
8	Model 1	0.634	

## Discussion

According to the results from the different tests described in these work and by contrasting them with the appraisals highlighted in [11], an increase in the number of original images does not fully guarantee a better result in terms of classification metrics. This was the case when we added “malignant” skin lesion images from the PH2 data set and obtained lower sensitivity than that what was obtained without PH2 images. Therefore, we are of the opinion that to get an improvement in classifier performance, if we include new images then these need to be of the same nature, i.e. two databases that have not been subject to the same pre-processing are not necessarily complementary to each other.

The performance of image classification systems can be affected by common transformations or alterations in the images, such as: rotation, flipping, zooming, contrast / brightness adjustment, etc. [30]. By introducing these transformations on the training data set, it is possible to increase the number of training samples while improving the robustness of the model against such transformations [31]. This data augmentation can act as a regularizer to prevent or reduce overfitting in neural networks [31–34] and improve performance in unbalanced data set [35]. In our work, the use of data augmentation techniques from the transformation of the original images, allowed to increase the training balance index (Malignant/Benign training ratio) and produced a positive impact that is used in the training stage, as shown in models 2–4.

Artificial intelligence (AI) algorithms focus primarily on the injury image for which they were designed. Although the evaluation of the surrounding tissue is not necessarily a predictor of the disease, it is key when making a diagnosis and in some cases, establishing a prognosis. In the case of actinic keratosis, chronic exposure to ultraviolet light causes damage to the tissue surrounding the main lesion, however, a human inspector still focuses on the main lesion, leaving aside this peripheral damage, which would improve the frequency of correct diagnoses by 32.5% [36]. In the case of melanoma, there may be involvement of the surrounding tissue, manifesting with metastatic lesions in transit, which are of poor prognosis or with satellite lesions that are locoregional cutaneous manifestations of dissemination as a consequence of embolization of tumor cells between the primary tumor and the regional lymph node; therefore, these lesions are highly predictive of lymphatic invasion and predict the development of disseminated disease [37, 38].

Morevoer, Tschandl et al. [36] showed that in Keratoses actinic detection, the surrounding area have clues for diagnosis, however each disease is described with different techniques and characteristics [39]. Thus, although the importance of perilesional skin involvement is clear, the objective of our study is to identify a lesion corresponding to melanoma and does not seek to establish a prognosis of the disease. Based on ABCDE [40] rule we can assume the study is focused only on the probable Melanoma lesion area because the relevant data to differentiate from other diseases is concentrated here, we make our model focus in this region by cropping the background before performing the classification task.

## Conclusions

The comparative analysis shown in "Discussion" section suggests a better performance for the proposed eVida M6 model over the methods previously proposed for the ISIC 2017 challenge. Automatic extraction of the region of interest within a dermatoscopic image suggests a significant improvement in classifier performance by eliminating pixels from the image that do not provide the classifier with lesion information.

The experiments in data augmentation described in "Results" section show that reducing data imbalance may be helpful to improve classification performance, but careful fine tuning is required, e.g. making training data perfectly balanced not necessarily results on a better model.

Finally, the eVida M6 model is a reliable predictor with an excellent balance between overall accuracy (0.904), sensitivity (0.820) and specificity (0.925). Within the set of tested models and also comparing with the best results reported in melanoma detection for the ISIC 2017 challenge, eVida M6 is an improvement in the state-of-the-art for automatic diagnosis of melanoma from dermatoscopic images.

## Data Availability

The datasets analysed during the current study are available in the ISIC repository, in https://www.isic-archive.com/#!/topWithHeader/onlyHeaderTop/gallery [24]/“ISIC Archive”/Accessed: 04-Jul-2020. The other dataset is available in the PH2 repository, in https://www.fc.up.pt/addi/ph2 database.html [28]/“ADDI—Automatic computer-based Diagnosis system for Dermoscopy Images” Accessed: 24-Jul-2019. Public access to the databases is open.

## Abbreviations

Mask R_CNN: Mask and Region-based Convolutional Neural Network
CNN: Convolutional Neural Network
ACS: American Cancer Society
ROI: Region Of Interest
ROC: Receiver Operating Characteristic
ISIC: International Skin Imaging Collaboration
ISBI: International Symposium on Biomedical Imaging
AUC_M: Area Under the RC Curve for Melanoma detection
ACC_M: Accuracy for Melanoma detection
SE_M: Sensitivity for Melanoma detection
SP_M: Specificity for Melanoma detection
